# Fluorescent CRISPR Adaptation Reporter for rapid quantification of spacer acquisition

**DOI:** 10.1038/s41598-017-10876-z

**Published:** 2017-09-04

**Authors:** Lina Amlinger, Mirthe Hoekzema, E. Gerhart H. Wagner, Sanna Koskiniemi, Magnus Lundgren

**Affiliations:** Department of Cell and Molecular Biology, Uppsala University, Uppsala, Sweden

## Abstract

CRISPR-Cas systems are adaptive prokaryotic immune systems protecting against horizontally transferred DNA or RNA such as viruses and other mobile genetic elements. Memory of past invaders is stored as spacers in CRISPR loci in a process called adaptation. Here we developed a novel assay where spacer integration results in fluorescence, enabling detection of memory formation in single cells and quantification of as few as 0.05% cells with expanded CRISPR arrays in a bacterial population. Using this fluorescent CRISPR Adaptation Reporter (f-CAR), we quantified adaptation of the two CRISPR arrays of the type I-E CRISPR-Cas system in *Escherichia coli*, and confirmed that more integration events are targeted to CRISPR-II than to CRISPR-I. The f-CAR conveniently analyzes and compares many samples, allowing new insights into adaptation. For instance, we show that in an *E*. *coli* culture the majority of acquisition events occur in late exponential phase.

## Introduction

CRISPR-Cas are prokaryotic adaptive immune systems that defend against *e*.*g*. bacteriophages^1^. They consist of a clustered regularly interspaced short palindromic repeats (CRISPR) array and CRISPR-associated (*cas*) genes that encode proteins required for immunity. CRISPR arrays are composed of repeats separated by short unique sequences called spacers, and are often preceded by a leader sequence^2–4^. Canonical CRISPR-Cas immunity is divided into three functional stages; adaptation, expression, and interference. During adaptation, new spacers acquired from *e*.*g*. phages and plasmids are integrated into the CRISPR array, enabling recognition of new targets^1^. During expression, the precursor CRISPR RNA (pre-crRNA) is processed and retained by Cas proteins, in some cases aided by additional factors^5, 6^. Finally, during interference the CRISPR RNA (crRNA) guides Cas proteins to complementary target sequences, which are subsequently cleaved or degraded^7, 8^.

Adaptation can be naïve, *i*.*e*. spacers are acquired from a sequence that is not already targeted by a spacer, which does not require the interference machinery^9^. The alternative is primed adaptation, where the interference machinery guides adaptation to a sequence that fully or partially matches a crRNA^10–12^. Frequency of spacer acquisition in a population may be affected by environmental regulatory cues and other factors^13–17^. The key proteins required for adaptation in all studied systems are Cas1 and Cas2. For details of the adaptation mechanism, see recent review^18^.

Here we report the development and characterization of a novel method for studying adaptation, fluorescent CRISPR Adaptation Reporter (f-CAR), using the type I-E CRISPR-Cas system of *Escherichia coli* MG1655^5^ as a model. MG1655 encodes two CRISPR loci, CRISPR-I carries 13 spacers and an adjacent set of *cas* genes whereas CRISPR-II (with six spacers) lacks neighboring *cas* genes^19^. The two CRISPR arrays differ in their respective leader sequences but have well conserved repeat sequences. The leader-proximal repeat sequence of the two CRISPR arrays only differs by one nucleotide. Naïve type I-E adaptation requires Cas1 and Cas2, 40–60 bp of the leader and one CRISPR repeat. Integration of a new 32 bp spacer results in duplication of the 29 bp leader-proximal repeat and the array is thus expanded by 61 bp^9^.

Several different methods to study CRISPR-Cas adaptation have been developed. In the most common approach, spacer integration is detected by PCR-amplification of the CRISPR arrays followed by gel electrophoresis, where the longer products generated from expanded arrays can be distinguished from the short products of unexpanded arrays^9, 10, 13, 20, 21^. However, detection of integration by PCR may be affected by amplification biases such as sequence preference and large differences in template abundance, and could result in false negative results^11^. PCR products or genomic DNA has also been analyzed by high-throughput DNA sequencing to quantify spacer integration^17, 22, 23^. PCR and sequencing methods assay populations of bacteria and require substantial sample processing before obtaining the results. Other assays are based on functionality, where new spacers mediate plasmid curing^10, 13^ or survival of phage infection^10, 20, 21, 24^. However, such methods do not detect non-functional or self-targeting adaptation events, as they may result in lack of phage protection or cytotoxicity^25^.

The f-CAR method allows rapid, easy-to-use, low-cost, sensitive, and quantifiable detection of adaptation. The method also enables detection of spacer acquisition in real-time and in single cells, which previously described methods do not. Our f-CAR system is based on a previously published system where spacer integration results in expression of a chloramphenicol resistance gene^26^, which allows for selection of cells that have acquired a spacer. We developed this system further by instead using a fluorescent read-out, enabling *in vivo* detection and quantification of adaptation. Here, the reporter is Yfp, referred to as Yfp-CAR, but f-CAR could be developed for use with other fluorescent reporter proteins. We characterize the developed reporter system and use it to investigate biological questions on type I-E CRISPR-Cas adaptation.

## Results and Discussion

### Design and construction of Yfp-CAR

**Figure 1:**
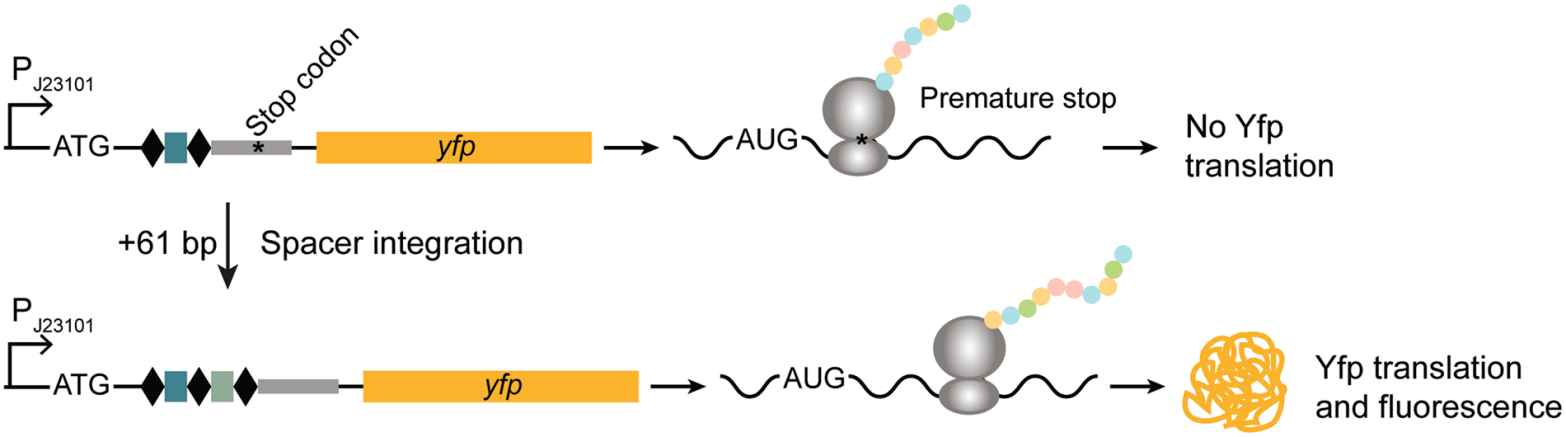
Construction of fluorescent CRISPR adaptation reporter. An unexpanded CRISPR array, consisting of two repeats (diamonds), one spacer (rectangle) and partial leader (grey) with in-frame stop codon, is inserted after a constitutive promoter and RBS (top). The reporter gene, here *yfp*, is in the +2 frame of the ATG and not translated with unexpanded array (top). Spacer acquisition adds 61 bp to the array (bottom), moving *yfp* into frame and stop codons out of frame, resulting in fluorescence.

To construct Yfp-CAR, the synthetic constitutive promoter pJ23101^27^, a Shine-Dalgarno sequence and a translational start codon (ATG) were placed upstream of a partial CRISPR array originating from either of the two CRISPR arrays in *E*. *coli* MG1655, CRISPR-I and CRISPR-II. These two constructs are referred to as Yfp-CAR^CR-I^ and Yfp-CAR^CR-II^. The arrays consist of 69 bp of the respective leader, including the sequence elements essential for adaptation^9, 28^, and one spacer flanked by two repeats. The *yfp* sequence lacking translational start signals was inserted downstream of the CRISPR array, such that upstream initiation results in out-of-frame translation (Fig. 1). In addition, translational stop codons (TAA) in the leader sequence are in frame with the ATG. For details and sequences of Yfp-CAR constructs see Supplementary Table S1. The reporter is designed for constitutive transcription but translation terminates within the leader sequence of the RNA. As adaptation lengthens the array by one 61 bp spacer-repeat unit, *yfp* is moved into frame and the stop codons in the leader out of frame, resulting in Yfp production and fluorescence (Fig. 1). Additional insertion events would move *yfp* out of frame again. The entire construct was inserted into the *galK* locus of the *E*. *coli* chromosome in a strain deleted for *cas* genes and both native CRISPR arrays. The latter was done to direct all integration events to Yfp-CAR and allow accurate quantification of spacer acquisition. The constructed strain cannot perform interference based on acquired spacers, ensuring that cells are not lost from the population due to self-targeting. Absence of Cascade and Cas3 prevents primed adaptation, hence all spacer acquisitions are naïve. Adaptation was enabled by plasmid-based expression of Cas1 and Cas2 from an IPTG-inducible T7 promoter^9^. Spacer acquisition assays were done in liquid culture with (or without) induction of Cas1 and Cas2 expression, followed by a period of regrowth with repression of *cas1* and *cas2* before measuring fluorescence (for details see Materials and Methods).

### Spacers can be inserted into Yfp-CAR

**Figure 2:**
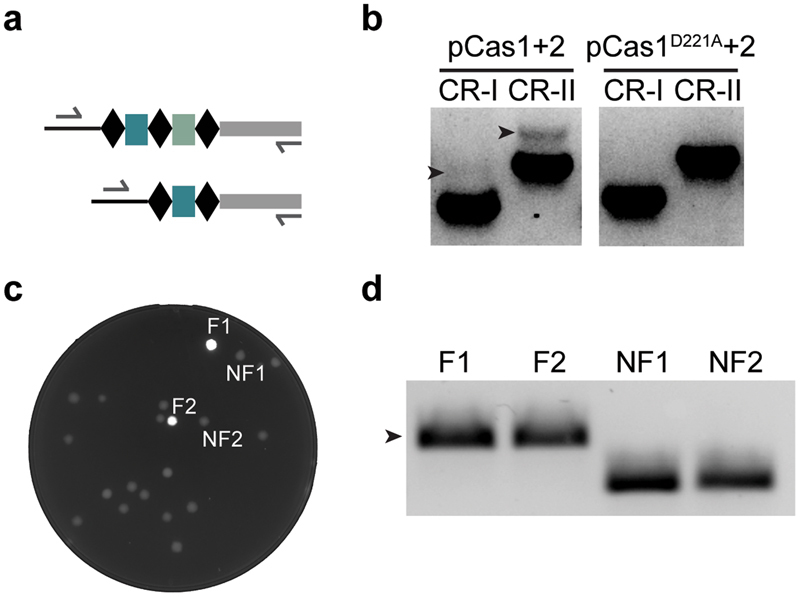
Functionality of Yfp-CAR. (**a**) PCR product length allows differentiation of expanded arrays (top) and unexpanded arrays (bottom). (**b**) PCR detection of expanded arrays (indicated with arrows) in Yfp-CAR^CR-I^ and Yfp-CAR^CR-II^ with active Cas1 and Cas2 (pCas1 + 2) or inactive Cas1 (pCas1^D211A^ + 2). (**c**) Detection of fluorescent colonies after spacer acquisition assay with Yfp-CAR^CR-II^. (**d**) PCR analysis of Yfp-CAR arrays in colonies indicated in (**c**). F, fluorescent; NF, non-fluorescent.

Yfp-CAR was first tested by performing a spacer acquisition assay followed by conventional detection of integration by PCR (Fig. 2a). When the Cas1-Cas2 complex was expressed, PCR products corresponding to unexpanded and expanded arrays were detected for both Yfp-CAR CRISPR arrays (Fig. 2b). This demonstrated that the reporter arrays are targeted for integration. As expected, no expansion of the arrays was detected with non-functional Cas1 (Fig. 2b).

### Spacer insertion into Yfp-CAR generates fluorescence

When cells from a spacer acquisition assay were transferred to solid media, fluorescent colonies could be observed after overnight incubation. As expected, the majority of colonies were non-fluorescent, but fluorescent colonies that maintained fluorescence after restreaking were readily detected (Figs 2c, S1 and S2). To confirm spacer integration in fluorescent colonies, we performed colony PCR on Yfp positive (Yfp+) and negative (Yfp−) colonies. All 27 tested fluorescent colonies had expanded CRISPR arrays whereas the 24 tested non-fluorescent colonies were unexpanded (Fig. 2d, supplementary Fig. S2, data not shown). We conclude that spacer integration leads to Yfp expression. Sequencing of PCR products from four selected Yfp+ colonies identified acquired spacers and their origin (Supplementary Fig. S3), demonstrating another useful application for the f-CAR. Three spacers were unambiguously derived from plasmid. The fourth spacer was from *lacI*, which is present on both plasmid and chromosome. However, the source is likely plasmid *lacI* as it is present in more copies than chromosomal *lacI*. The sequenced spacers were all different and corresponded to individual spacer integration events that likely occurred in the liquid culture before plating.

To further characterize adaptation of the Yfp-CAR CRISPR arrays, a spacer acquisition assay was performed and PCR-amplified arrays from the experiment were analyzed by high throughput sequencing. Out of a total of 28,305 and 28,139 array sequences, 34 and 197 demonstrated expanded arrays for Yfp-CAR^CR-I^ and Yfp-CAR^CR-II^, respectively (Table 1). Previous analyses indicated that approximately 95% of integrated spacers should be 32 bp^19^. Spacers with different length would not move the reporter gene into frame, but we detect only one spacer of aberrant length (33 bp) that, incidentally, also contained a 1 bp deletion in a repeat so the array was still extended by a total of 61 bp. In addition, newly incorporated spacers can contain in-frame stop codons resulting in translation termination and absence of fluorescence. We found that 53% of spacers inserted into Yfp-CAR^CR-I^ and 68% into Yfp-CAR^CR-II^ had no in-frame stop codons in any of the ten possible positions (Table 1), and would therefore permit Yfp expression after adaptation. These numbers correlate well with the calculation that 59% of spacer integration events should allow f-CAR fluorescence, on average^26^. It should be noted that the frequency of fluorescent cells (here Yfp+) is thus not equal to the absolute adaptation frequency. Relative differences are, however, still accurately measured. In line with previously observed bias in spacer acquisition from plasmids rather than the chromosome in *E*. *coli* ^9^, the majority of acquired spacers were derived from the plasmid expressing Cas1 and Cas2, and only 6 and 18 spacers originating from the chromosome for Yfp-CAR^CR-I^ and Yfp-CAR^CR-II^, respectively (Table 1, Supplementary Table S2.) As mentioned above *lacI* spacers likely originated from the plasmid and are listed as such. Three spacers did not map to the plasmid or the chromosome, and could be derived from sequences present in the organism but not represented in the available plasmid and genome sequences. The spacers origins were distributed over the entire plasmid and indicated no strand bias (Supplementary Fig. S4). Most sequenced spacers were unique, corresponding to individual acquisition events, and not amplifications of spacers due to *e*.*g*. growth advantage or PCR bias.

**Table 1:** Summary of analysis of expanded arrays by SMRT sequencing.

Sample	Total no. of sequences	Expanded sequences	32 bp spacers	Spacers without in-frame stop codons	Expanded sequences supporting Yfp fluorescence	Unique spacers	Genome targeting spacers
Yfp-CAR^CR-I^	28,305	34	34	18	53%	33	6
Yfp-CAR^CR-II^	28,139	197	196	133	68%	170	18

Spacer acquisition assay using Yfp-CAR^CR-I^ and Yfp-CAR^CR-II^ was analyzed by SMRT sequencing of the CRISPR arrays in the two populations.

### Yfp-CAR accurately and sensitively quantifies cells with expanded arrays

**Figure 3:**
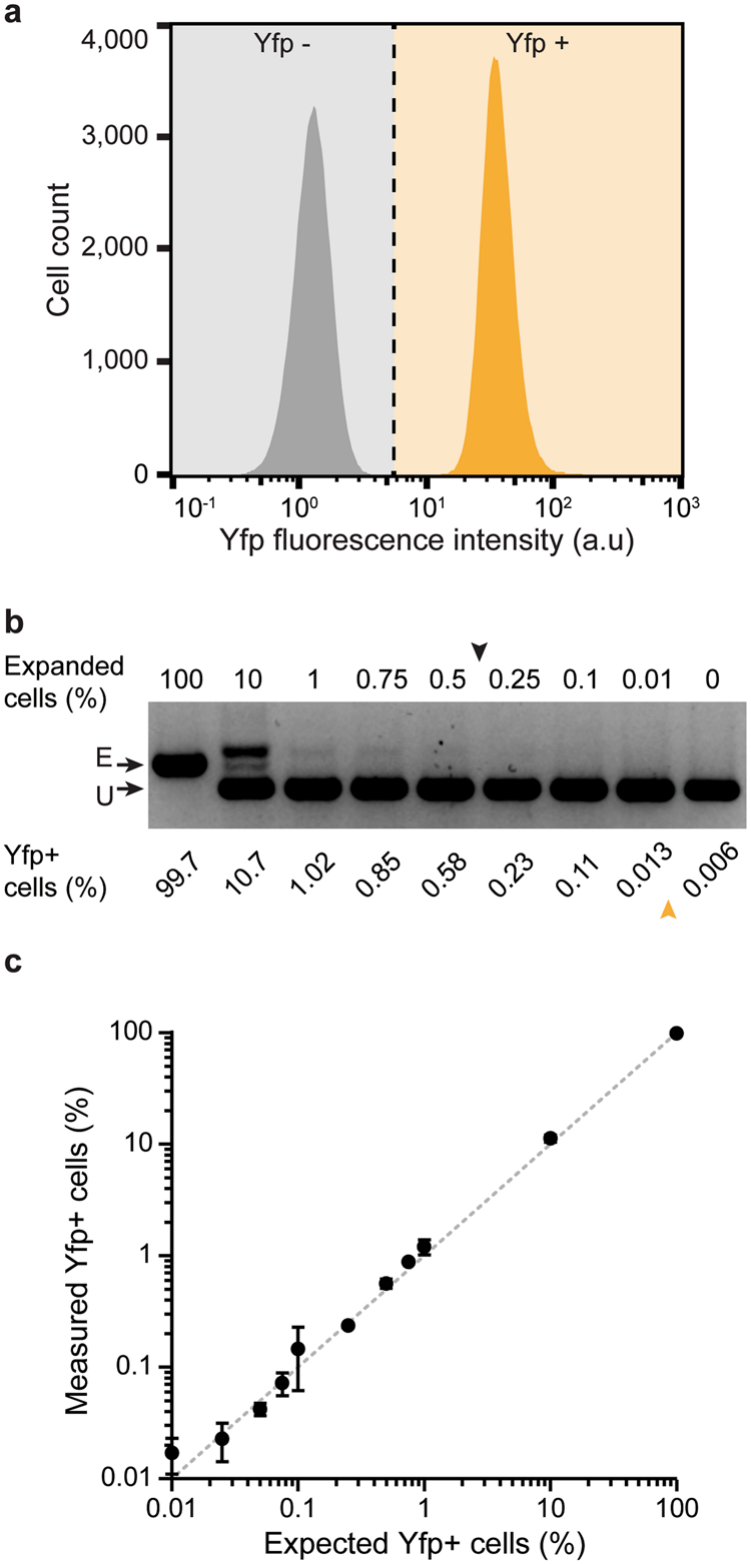
Sensitivity and accuracy of detection of spacer integration using Yfp-CAR. (**a**) Flow cytometry fluorescence analysis of control cells with unexpanded (grey) or pre-expanded (yellow) CRISPR array, both with inactive Cas1 (pCas1^D221A^ + 2). Dashed line indicates gates set so <0.01% Yfp+ events is detected for control cells with unexpanded array. (**b**) PCR detection of expanded CRISPR arrays in mixtures of cells with pre-expanded (E) or unexpanded (U) Yfp-CAR^CR-I^. Arrows indicate detection limit for PCR (black arrow, top) and flow cytometry (yellow arrow, bottom). (**c**) Detection of Yfp+ cells by flow cytometry plotted against expected percentage of Yfp+ cells in mixtures of cells with expanded and unexpanded arrays. The expected ratios are indicated by the dotted line. Error bars: SD, N = 4.

To determine the detection limit and accuracy of Yfp-CAR, cells from a designed pre-expanded control strain (Yfp+) were mixed at different ratios with cells carrying the unexpanded array (Yfp−) and analyzed by PCR and flow cytometry (Fig. 3). For all flow cytometry analysis, gates were set so that less than 0.01% positive events were detected in the strain with an unexpanded array and a non-functional Cas1 (Fig. 3a). By PCR, spacer integration could be detected in a sample with 0.5% cells with expanded arrays, but not with 0.25% (Fig. 3b). This correlates well with the previously reported PCR detection limit of 0.4% expanded arrays^9^. In contrast, flow cytometry reproducibly detected expanded arrays when present in as few as 0.05% cells (Fig. 3c). Furthermore, samples with 0.5, 0.75 and 1% expanded arrays generated PCR bands of similar intensity, obscuring quantitative differences between these samples, whereas the expected percentages could be reproducibly measured by flow cytometry (Fig. 3b,c). We conclude that Yfp-CAR is at least 10-fold more sensitive than PCR-based assays, and that reliable quantification of adaptation events can be obtained even at low frequencies.

### Quantification and detection of adaptation using Yfp-CAR

**Figure 4:**
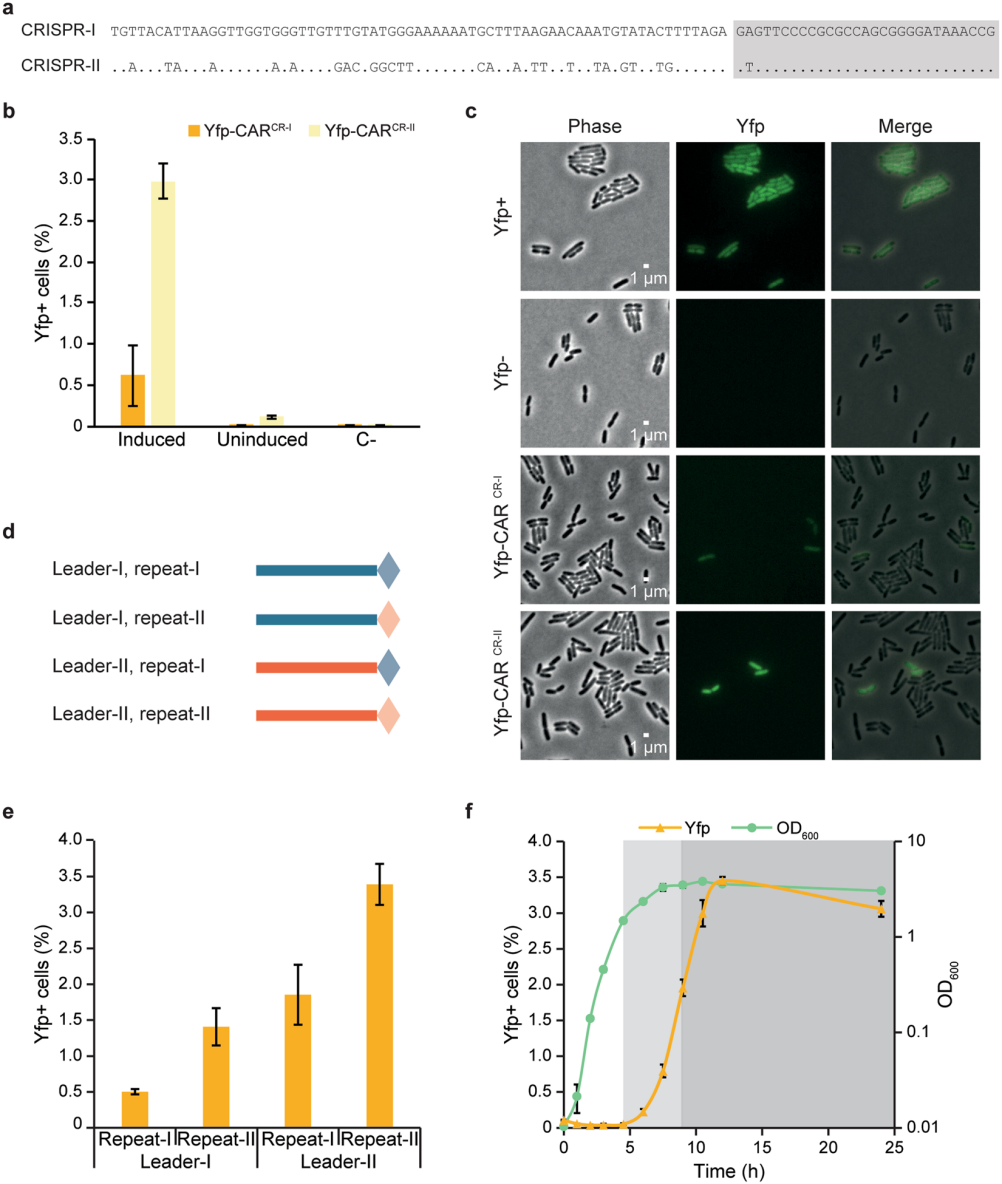
Quantification of adaptation using Yfp-CAR. (**a**) Alignment of the partial leader and leader-proximal repeat of the two CRISPR arrays in MG1655 used in this study. Identical bases are indicated by · in CRISPR-II. Repeat is highlighted in grey. (**b**) Flow cytometry detection of Yfp + cells in strains with Yfp-CAR after spacer acquisition assay and regrowth with or without induction of Cas1-Cas2 expression. Negative control strain (C-) with unexpanded Yfp-CAR and pCas1^D221A^ + 2 shown for comparative purpose. Error bars: SD. N = 6 (Yfp-CAR^CR-I^), N = 3 (Yfp-CAR^CR-II^). (**c**) Imaging of indicated strains after spacer acquisition assay and subsequent regrowth with phase contrast and fluorescence microscopy. Scale bar is 1 µm. (**d**) Graphical representation of the minimal CRISPR arrays used in (e), consisting of partial leader (line) and one repeat (diamond) from CRISPR-I (blue) or CRISPR-II (orange). (**e**) Flow cytometry quantification of integration into minimal Yfp-CAR with leader sequences and repeat sequences in the indicated combinations after spacer acquisition assay and subsequent regrowth. Error bars: SD. N = 3. (**f**) Time course of adaptation using Yfp-CAR^CR-II^ with induction of Cas1 and Cas2 expression at t = 0. Samples were withdrawn at the indicated time points and Yfp + cells were quantified by flow cytometry after a short regrowth. Non-shaded area: exponential phase (t = 0 to t = 4.5 h), light grey area: late exponential phase (t = 4.5 h to 9.5 h), dark grey area: stationary phase. Error bars: SD. N = 3.

Spacer integration in Yfp-CAR^CR-I^ and Yfp-CAR^CR-II^ after spacer acquisition assays was quantified by flow cytometry. With Yfp-CAR^CR-I^, 0.6% of the cells were fluorescent (Fig. 4b) whereas 3.0% of the cells with Yfp-CAR^CR-II^ were fluorescent (Fig. 4b), suggesting that spacer integration is about five times more frequent in Yfp-CAR^CR-II^ than in Yfp-CAR^CR-I^. Congruent with this, a 5.8-fold preference for adaptation into Yfp-CAR^CR-II^ was observed by comparing the number of expanded sequences obtained by high throughput sequencing (Table 1). The qualitative difference is in line with a previous report where more integration events were detected in CRISPR-II than in CRISPR-I^10^. Adaptation could also be detected in Yfp-CAR^CR-II^ in a strain with the two wildtype CRISPR arrays and the endogenous chromosomal *cas* genes (data not shown), demonstrating that integration occurs into Yfp-CAR even in the presence of native arrays. It should be noted that the endogenous *E*. *coli cas* genes are silenced by H-NS^29^, so potentially lethal targeting of chromosome, or plasmid with selection marker, by CRISPR-Cas (which would preclude detection of spacer acquisition) does not occur under these conditions.

Without induction of Cas1 and Cas2 proteins, the percentage of fluorescent cells was dramatically reduced, but still above that in the negative controls (Fig. 4b). This is probably due to low-level leaky expression of Cas1 and Cas2.

Cells from assayed strains were also examined by microscopy. As expected, fluorescent cells were detected in a fraction of the population (Fig. 4c). Spacer integration in cells carrying Yfp-CAR^CR-II^ was also investigated by manual time-lapse microscopy. Analysis after 24 h of Cas1 and Cas2 protein expression detected the presence of fluorescent cells. These cells continued to produce fluorescent offspring (Supplementary Fig. S5), suggesting that no new spacers are integrated during imaging. Some cells were non-fluorescent at the beginning of regrowth but later became fluorescent (Supplementary Fig. S5). They likely represent integration events that occurred late in stationary phase, without enough time for detectable Yfp expression before initial imaging, but could also correspond to real-time observations of spacer integration.

### Both leader and repeat sequences affect adaptation frequency

While the leader sequence of CRISPR-I and CRISPR-II differ substantially, their leader-proximal repeats display only one nucleotide difference (Fig. 4a). To investigate the sequence elements affecting adaptation frequencies and causing the observed differences in integration into Yfp-CAR^CR-I^ and Yfp-CAR^CR-II^, minimal Yfp-CAR reporters were constructed with CRISPRs consisting only of the leader sequence and the leader-proximal repeat (Fig. 4d). Four combinations of leader and repeat sequences were tested for integration (Fig. 4d,e). Higher integration frequencies were observed for Yfp-CAR^L-II, R-II^ (corresponding to CRISPR-II) than for Yfp-CAR^L-I, R-I^ (corresponding to CRISPR-I), similar to previous results. Average adaptation frequencies into the minimal reporter arrays were comparable to those of the longer arrays, 0.5% for Yfp-CAR^L-I, R-I^ vs. 0.6% Yfp-CAR^CR-I^ and 3.4% Yfp-CAR^L-II, R-II^ vs. 3.0% for Yfp-CAR^CR-II^ (*cf*. Fig. 4b and e), indicating that arrays with only one repeat are as functional as the longer arrays. Interestingly, the arrays with combinations of leader and repeat from different arrays exhibited intermediate adaptation frequencies (Fig. 4e), suggesting that both leader and repeat sequences contribute to adaptation efficiency. The sequence of the leader-repeat boundary has previously been shown to affect spacer integration frequencies, both *in vitro* and *in vivo* ^28^, but within this region only one base pair differs between the arrays. This single base pair difference in the repeat sequence decreases adaptation frequency by almost half, possibly due to a critical position in the insertion site for the new spacer^28^. The leader sequences are less conserved (Fig. 4a) and further studies are required to elucidate the sequences within the leader that affect adaptation frequencies.

### New spacers are primarily integrated during late exponential phase

As a further demonstration of the utility of f-CAR, the percentage of Yfp fluorescent cells was monitored over time during an acquisition assay. This time course assay addressed whether adaptation preferentially occurs during a specific growth phase. As integration is more frequent into CRISPR-II than into CRISPR-I, Yfp-CAR^CR-II^ was used. Samples were taken at regular time intervals, and the percentage of Yfp+ cells was measured after a short regrowth, thus cancelling out differences in fluorescence due to *e*.*g*. different Yfp-CAR promotor activity or Yfp maturation in the different growth phases. During early exponential phase (Fig. 4f, white area), the percentage of Yfp+ cells remained unchanged, while it rapidly increased during late exponential phase (Fig. 4f, light grey area). The fraction of Yfp+ cells continued to increase in early stationary phase and plateaued during late stationary phase (Fig. 4f, dark grey area) to the same level observed in the previous acquisition assays with Yfp-CAR^CR-II^ (Fig. 4b and f). We conclude that, under the experimental conditions used, adaptation occurs during late exponential/early stationary phase, with very little or no adaptation during exponential growth and late stationary phase. Since Cas protein expression was induced we cannot exclude that the induction kinetics affect the observed growth phase dependence. It is however tempting to speculate that chromosome topology, replication or presence/absence of other factors may also play a role. As an example the IHF protein, which is required for adaptation^28^, has been demonstrated to be most abundant in late exponential phase^30^.

## Conclusion and Outlook

The fluorescent reporter for adaptation (f-CAR) described in this communication enables real-time, quantitative detection of acquisition events by the CRISPR-Cas system in *E*. *coli*. The method allow several options for measuring spacer integration by fluorescence; in single cells by *e*.*g*. flow cytometry or microscopy as well as in colonies. The system could be developed for other organisms and different types of CRISPR-Cas systems as long as the repeat-spacer unit is not (always) a multiple of three base pairs. Other possible developments are addition of a full set of *cas* genes to the system which, together with expression of pre-crRNA, could allow investigation of interference and primed adaptation. Also, detection of multiple spacer insertions could be achieved by further shifting the frame of *yfp* or by inserting multiple fluorescent reporter genes in different frames.

To demonstrate this read-out system’s advantageous properties to address CRISPR-Cas biology, we performed several experiments. We confirm that adaptation is more frequent for CRISPR-II than for CRISPR-I, and demonstrate that both the leader and repeat sequences of CRISPR-II contribute to it being more favored for insertion than CRISPR-I. Furthermore, we show that adaptation primarily takes place in late exponential/early stationary phase.

In conclusion, f-CAR is a fluorescent reporter system that provides new and significantly improved possibilities for detection and quantification of CRISPR-Cas adaptation. The f-CAR method will hopefully assist further investigations of both fundamental aspects and details of the adaptation process of CRISPR-Cas immune systems.

## Methods

### Reagents and growth conditions

Routine growth of *E*. *coli* was done in LB (5 g/L yeast extract, 10 g/L NaCl, 10 g/L tryptone), supplemented with the appropriate antibiotics (50 mg/L kanamycin, 100 mg/L ampicillin, 50 mg/L streptomycin, 15 mg/L chloramphenicol or 15 mg/L tetracycline). Media components were from Oxoid. All other chemicals, and all oligonucleotides, were from Sigma-Aldrich.

### Strains and plasmids

For all experiments, a derivative of MG1655 deleted for all *cas* genes and both CRISPR arrays was constructed. The *cas* genes and CRISPR-I were deleted by Lambda Red recombination^31^, through insertion of a kanamycin cassette amplified from pKD4^31^ with primers LML009 and LML010 (Supplementary Table S3). The kanamycin cassette was subsequently removed using the Flp recombinase encoded by pCP20 as previously described^32^, creating a marker-less deletion of *cas3*-CRISPR-I. CRISPR-II was deleted by scar-less mutagenesis using the *kan-sacB* cassette^33^. Briefly, the CRISPR-II array was replaced by a *kan-sacB* cassette by Lambda Red recombination and subsequent kanamycin selection. Next, a single-stranded DNA oligonucleotide, LA157, was used to remove the *kan-sacB* cassette in a second recombination step. Sucrose toxicity in cells expressing SacB was used to select for cells that had successfully replaced the *kan-sacB* cassette with the provided oligonucleotide.

Cas1 and Cas2 were expressed from pCas1 + 2^9^ (Supplementary Table S4), where Cas1 and Cas2 are cloned under an IPTG-inducible T7-promoter. Cas1 with a point-mutation inactivating its DNase activity was used as a negative control; pCas1^D221A^ + 2 (Supplementary Table S4)^9^. The gene encoding the T7 polymerase was inserted in the *araBAD*-operon by P1 mediated transduction from MLS640. MLS640 is a BW25113 derivative, constructed by insertion of the T7 polymerase and tetracycline resistance gene in the *araBAD*-locus by Lambda Red recombination. The *T7 polymerase* and *tetR* were amplified from BL21AI using LA120 and LA121 primers. Primers and overhangs were chosen so that the intergenic region between *araC* and the *araBAD* promoter was reconstituted in the constructed BW25113-strain, but the *araBAD* operon was not. All relevant primers and DNA oligonucleotides can be found in Supplementary Table S3. All relevant strains and plasmids can be found in Supplementary Table S4.

### Construction of Yfp-CAR

A *yfp* gene (sYFP2, Genbank KM018300^34^) was cloned after the CRISPR-array from pCSIR-T^26^, which corresponds to parts of CRISPR-I. The *yfp* was cloned out of frame (+2) of the start codon (ATG) with the CRISPR-I array between the ATG and the *yfp*. This also places a stop codon (located in the leader) in frame with the start codon, before the *yfp*. The partial array includes 69 bp of the leader, two repeats, one spacer and one partial spacer. The sequence is identical to CRISPR-I in MG1655 except for a point mutation in the leader (−42 from the first repeat) to prevent an in-frame stop codon after spacer integration^26^. To improve the fluorescence signal, an unstructured linker was placed between the CRISPR array and the *yfp*. This construct was PCR amplified with primers LA151 and LA152 and integrated in the *galK* locus after the constitutive, synthetic promoter J23101^27^ replacing a previously integrated *rfp* in such a way that the ATG of the pCSIR-T construct was substituted with that of the *rfp* gene, creating strain MLS904 (Fig. 1). The CRISPR array was placed in the opposite direction of transcription to prevent crRNA production which could interfere with the translation of Yfp, *e*.*g*. if the construct is combined with Cascade which would bind the repeat of the crRNA. An Yfp-positive control for Yfp-CAR^CR-I^ was constructed by inserting a 61 bp random sequence without stop codons in the first spacer (strain MLS902). To create Yfp-CAR^CR-II^ with the CRISPR-II array, a *kan-sacB* cassette was inserted between the translational start codon and *yfp* in strain MLS904 replacing the CRISPR-I array (strain MLS988). The *kan-sacB* cassette was subsequently replaced with 69 bp of the leader and a repeat-spacer-repeat unit from CRISPR-II, PCR amplified from MG1655 (primers LA164 and LA166) as described above, creating a scar-less replacement of CRISPR-I by CRISPR-II in Yfp-CAR (strain MLS989). A positive control for Yfp-CAR^CR-II^ was created by adding one base pair to the inserted sequence thereby moving the *yfp* into frame (primers LA165 and LA166, creating strain MLS990). Sequences of the Yfp-CAR constructs can be found in Supplementary Table S1.

To construct the minimal CRISPR arrays corresponding to leader and a single repeat, with all combinations of the sequences, the leader and repeat was amplified from MLS904 with pCas1^D221A^ + 2 and MLS989 with pCas1^D221A^ + 2 using primers LA168 and LA171 or LA172, with LA171 corresponding to a CRISPR-I repeat, and LA172 corresponding to a CRISPR-II repeat. The resulting PCR products were used as template in a second PCR with LA168 and LA173 to add the overhangs needed for insertion by Lambda Red recombination. The minimal CRISPR arrays were inserted between the ATG and the *yfp* in MLS988 by Lambda Red recombination as described for CRISPR-II above. Minimal CRISPR constructs were defined by leader-repeat combinations, Yfp-CAR^L-I, R-I^ (MLS1000), Yfp-CAR^L-II, R-I^ (MLS1001), Yfp-CAR^L-I, R-II^ (MLS1002), and Yfp-CAR^L-II, R-II^ (MLS1003). Sequences of the minimal Yfp-CAR array constructs can be found in Supplementary Table S1.

### Spacer acquisition assay

For spacer acquisition assays, overnight cultures, made in LB with 50 mg/L streptomycin, were diluted 1:600 in LB with 50 mg/L streptomycin and 0.1 mM IPTG and 0.2% arabinose to induce Cas protein expression. Assay was performed at 37 °C for 24 h. To allow for expression and maturation of Yfp from late integration events, the cultures were subsequently diluted 1:100 in LB with 0.2% glucose, to repress further Cas protein expression, and grown for 5 h at 37 °C. After 5 h, samples were taken for flow cytometry for single-cell detection of integration as well as for PCR for detection of integration in the whole population (see below).

For time course experiment, overnight cultures were made in LB with 50 mg/L streptomycin, 1% glucose and 0.1 M potassium phosphate buffer in order to prevent leaky expression of Cas1 and Cas2 which may cause premature spacer integration. Overnight cultures were diluted as before and, at indicated time points, samples were taken and OD_600_ was measured. The samples were centrifuged for 2 minutes to pellet cells and cells were resuspended in LB with 0.2% glucose to approximately the same OD_600_ (0.025) for all time points. Regrowth was done for 1.5 h at 37 °C. After regrowth, cells were pelleted by centrifugation for 2 min, and resuspended in ice cold, sterile filtered phosphate-buffered saline (PBS). Samples were kept on ice for the duration of the time course and integration was detected as Yfp + cells by flow cytometry as described below.

### Spacer integration detection by PCR

For PCR, 500 µl of culture was pelleted and cells were resuspended in 100 µl sterile water. The sampled volume was adjusted according to OD_600_ to sample approximately the same number of cells for all assayed cultures. For spacer integration assays, OD_600_ were 3.5–4.5 after 24 h of growth. Cells were lysed by boiling 10 min at 95 °C. Cell debris was pelleted and 5 µl of culture supernatant was used for a 50 µl PCR reaction using DreamTaq Green DNA polymerase (Thermo Fisher Scientific) and primers amplifying the CRISPR array (for Yfp-CAR^CR-I^ : LA112 and LA007, for Yfp-CAR^CR-II^ : LA112 and LA168; Supplementary Table S3). 10 µl PCR product was analyzed on a 1.5% agarose Tris-borate-EDTA (TBE) gel with SYBR safe (Thermo Fisher Scientific) visualized in ChemiDoc^TM^ MP with Image lab v. 4.0 software (Bio-Rad) using the pre-set SYBR Safe application (Excitation: UV trans illumination, Emission: Standard filter).

### Spacer integration detection in colonies

Cells were plated on LA (5 g/L yeast extract, 10 g/L tryptone, 10 g/L NaCl, 15 g/L agar) with 0.2% glucose directly after the spacer acquisition assay and grown overnight at 37 °C. Fluorescent colonies were visualized in ChemiDoc^TM^ MP with Image lab v. 4.0 software (Bio-Rad) using Alexa 546 settings (excitation: blue epi illumination, emission: 605/50 filter). For selected colonies, the CRISPR array was investigated by colony PCR, using the same primers as above, and Illustra PuRe Taq Ready-to-Go PCR beads (GE Healthcare). The same colonies were re-streaked to confirm the fluorescent phenotype. 5 µl PCR product was analyzed on 1.5% agarose TBE gel with SYBR safe (Thermo Fisher Scientific) and visualized in ChemiDoc^TM^ MP (Bio-Rad) as described above.

### Single cell detection of spacer integration by flow cytometry

For single cell analysis of spacer integration events, flow cytometry was performed using a MACSQuant VYB (Miltenyi Biotec). Samples were appropriately diluted in sterile filtered PBS and loaded in a 96-well polystyrene plate for analysis. SYFP2 was excited with a blue laser (488 nm; bandpass filter 525/50 channel B1). 100,000 events were recorded for each sample. Data were acquired with the MACSQuantify^TM^ Software (Miltenyi Biotec) and processed with FlowJo Software (FlowJo, LLC).

Events were gated for bacterial cells using side scatter measurements; these were subsequently classified as Yfp positive or negative by bifurcating the Yfp channel. The Yfp signal was always gated in such a way that there were <0.01% positive events in the negative control (strain MLS904 or MLS989 with pCas1^D221A^ + 2). The number of Yfp positive events for three (Yfp-CAR^CR-II^) or six (Yfp-CAR^CR-I^) biological replicates were averaged, error bars indicate standard deviation.

### High-throughput sequencing of acquired spacers

Yfp-CARs were sequenced after a spacer acquisition assay using Single Molecule Real Time (SMRT) sequencing. To create the PCR products used for SMRT sequencing, cell lysate from a representative spacer integration assay of Yfp-CAR^CR-I^, Yfp-CAR^CR-II^ was used as template. Cell lysate preparation was done as described above. PCR reactions were performed using Phusion Hot Start Flex DNA polymerase (New England Biolabs) for 35 rounds of amplification. The same primer pair, LA112 and LA168, was used for both Yfp-CARs to minimize potential amplification bias caused by primer differences. PCR product was purified using AMPure PB beads (Pacific BioSciences). Templates were prepared using maximum recommended amount of adapters and sequenced on a PacBio RS II sequencer (Pacific BioSciences) using one SMRT cell per sample. Circular consensus sequencing (CCS) algorithm was used to generate sequences, and information about newly acquired spacers was extracted.

### Test of detection limit

To examine the detection limit of spacer integration by PCR and flow cytometry, we performed a dilution test using the Yfp-CAR^CR-I^ pre-expanded Yfp positive control (MLS902) and the strain with unexpanded array (MLS904), both with the inactive Cas1 to prevent integration of new spacers. Overnight cultures were diluted 1:100 in LB with 0.2% glucose and grown for 5 h at 37 °C. OD_600_ was adjusted to 4 for both cultures before serial dilution of the pre-expanded control in the strain with unexpanded CRISPR array. Samples were taken for analysis by flow cytometry and 500 µl was used for spacer integration PCR as described above. In the same way, three more dilution series were made using the Yfp-CAR^CR-II^ positive control (MLS990) and unexpanded strain (MLS989), again with pCas1^D221A^ + 2. Overnight cultures were back diluted as above and the fresh cultures were diluted to an OD_600_ of 4. The Yfp positive control strain (MLS990) was serially diluted in the unexpanded strain (MLS989) in three independent series and analyzed by flow cytometry.

Gating of cells after flow cytometry was done as described above. The numbers of Yfp positive events from the four dilution series (one with Yfp-CAR^CR-I^ and three with Yfp-CAR^CR-II^) were averaged and the standard deviation was calculated.

### Fluorescence microscopy

After spacer acquisition assay and subsequent regrowth, samples were taken for microscopy. Cells were imaged using a Nikon Ti-Eclipse inverted microscope for phase contrast and Yfp fluorescence using a 20 ms capture time. Images were captured using a sCMOS camera (Andor, UK) with a Nikon CFI Plan Apo Lambda 60X Oil objective and all images were processed the same way using Adobe Photoshop CS6 software for overlays.

For real-time imaging (Supplementary Fig. S5) cells were placed on a 1% agarose pad supplemented with 1x LB directly after spacer acquisition assay, and images were acquired at three time points (0 h, 1 h, and 3 h) as described above.

## Electronic supplementary material

Supplementary material

Supplementary table
