# Supplementary material for: Fluorescent CRISPR Adaptation Reporter for rapid quantification of spacer acquisition
Source: Sci Rep. 2017 Sep 4;7:10392. doi: 10.1038/s41598-017-10876-z (PMC5583386; doi:10.1038/s41598-017-10876-z)
Supplement: Supplementary file 1 — Supplementary material [file 41598_2017_10876_MOESM1_ESM.pdf]

Fluorescent CRISPR Adaptation Reporter for rapid quantification of spacer acquisition

Lina Amlinger, Mirthe Hoekzema, E. Gerhart H. Wagner, Sanna Koskiniemi and Magnus Lundgren

Supplementary material

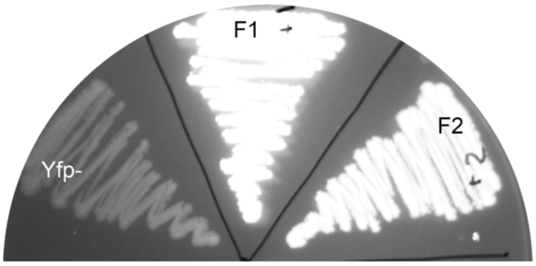

Supplementary figure S1. Confirmation of fluorescence of colonies obtained for Yfp-CAR^{CR-II}. Re-streaks of fluorescent colonies in Fig. 2c to confirm fluorescent phenotype. Fluorescence was visualized after overnight incubation at 37°C.

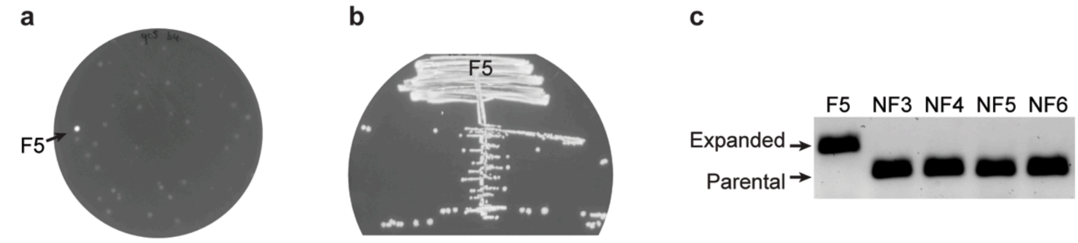

Supplementary figure S2. Investigation of fluorescent and non-fluorescent colonies from acquisition assay using Yfp-CAR^{CR-I}. (**a**) Cells were plated on LA+0.2% glucose after spacer acquisition assay. Fluorescence was visualized after incubation overnight. (**b**) Re-streak of fluorescent colony (F5) from (a). (**c**) Colony PCR of fluorescent and non-fluorescent colonies from Yfp-CAR^{CR-I}. F, fluorescent, NF, non-fluorescent colonies.

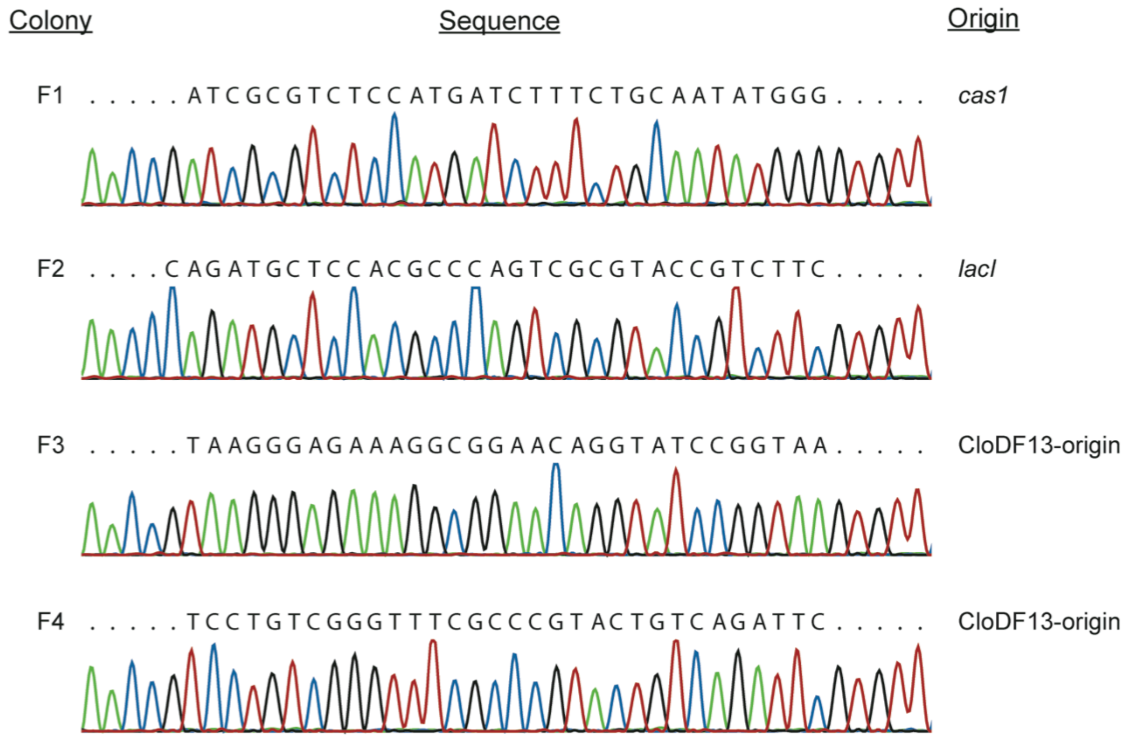

Supplementary figure S3. Chromatograms and sequences of acquired spacers. Four fluorescent colonies obtained after acquisition assay with Yfp-CAR$^{CR\text{-}II}$ were analyzed by PCR and the purified PCR-products were sequenced. All colonies were confirmed to be fluorescent by re-streaks prior to sequencing. Sequences F1 and F2 are from the indicated colonies and PCR-fragments in Fig. 2c and 2d.

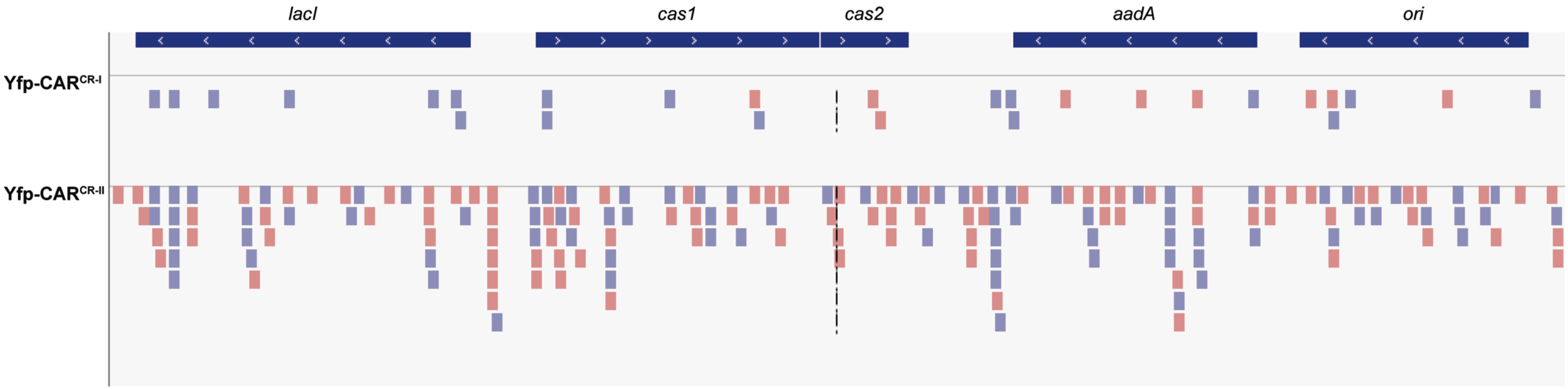

Supplementary figure S4. Spacers acquired from pCas1+2 mapped onto the plasmid. Spacers acquired during a spacer acquisition assay and analyzed by SMRT sequencing mapped onto the plasmid pCas1+2. The genes on the plasmid are indicated. Spacers of same color would generate crRNA that would target the same strand of plasmid DNA.

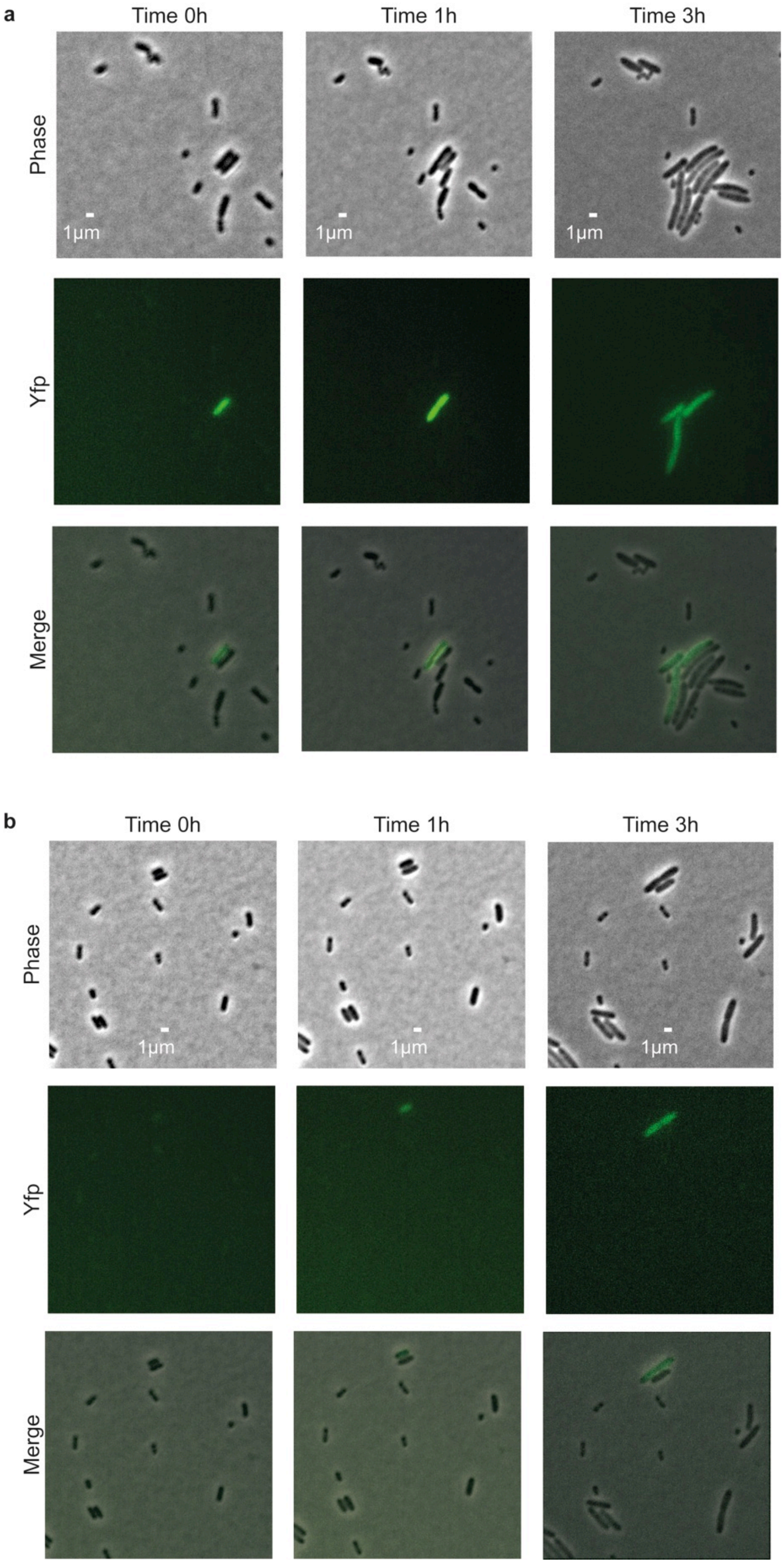

Supplementary figure S5. Time-lapse microscopy of Yfp-CAR^{CR-II} after 24 h of Cas1-2 expression. The same positions were imaged with phase contrast and for Yfp fluorescence directly (0 h), 1 h, and 3 h after a spacer acquisition assay. (**a**) A fraction of cells was fluorescent already at 0 h and gave rise to fluorescent offspring after division. (**b**) Another fraction of cells was not fluorescent at 0 h, but gained fluorescence during the experiment.

Supplementary table S1. Sequences of Yfp-CAR constructs used in this study.

J23101 promoter is highlighted in cyan and the linker sequence in grey. The translational start codon is in blue. The CRISPR arrays are separated by spaces from the sequences identical in all constructs. Note that the CRISPR arrays are cloned in the opposite direction of transcription. CRISPR repeats are highlighted in green and the leader is underlined with the in-frame stop codons marked in red. The mutation in the leader of CRISPR-I is highlighted in magenta (mutation introduced in pCSIR-T as described in Díez-Villaseñor, C., Guzmán, N.M., Almendros, C., García-Martínez, J. & Mojica, F.J. RNA Biol 10, 792-802 (2013)). The first codon of *yfp* is highlighted in yellow. Inserted sequence to create the pre-expanded control of Yfp-CAR$^{\text{CR-I}}$ is in green italic font, as is the inserted base to move the *yfp* into frame in Yfp-CAR$^{\text{CR-II}}$.

Construct	Sequence
**Yfp-CAR$^{\text{CR-I}}$ (MLS904)**	TTTACAGCTAGCTCAGTCCTAGGTATTATGCTAGCTACTAGAGAAAGAGGAGAAATACTAG**ATG**ACCATGATTACGCCAAGCTTGGTACCGAGCTCGGA<br>TCCACTAGTAACGGCCGCCAGTGTGCTGGAATTCGGCT TCACCTTTGGCTTCGGCTGCGGTTTATCCCCGCTGGCGCGGGGAACTCTGCGTGAGCG<br>TATCGCCGCGCGTCTGCGAAAGCGGTTTATCCCCGCTGGCGCGGGGAACTCTCTAAAAGTATACATTTGTTCT**TAA**AGCATTTTTCCCATACAAACAA<br>CCCACCAACCTTAATGTAACA AAAGCCGGTTCAGGGTCAGGTTCTGGTTCTGGAAGCGTT
**Yfp-CAR$^{\text{CR-I}}$ YFP positive control (pre-expanded array) (MLS902)**	TTTACAGCTAGCTCAGTCCTAGGTATTATGCTAGCTACTAGAGAAAGAGGAGAAATACTAG**ATG**ACCATGATTACGCCAAGCTTGGTACCGAGCTCGGA<br>TCCACTAGTAACGGCCGCCAGTGTGCTGGAATTCGGCT TCACCTTTGGCTTCGGCTGCGGTTTATCCCCGCTGGCGCGGGGAACTCTGCGTGAGCG<br>TATCGC***TGATTGGAGTTTCAGTCCGATACCTGCTTCGTTGAGAACTCACAATTTTACAACTGGGGAC***CGCGCGTCTGCGAAAGCGGTTTATCCCCGCTG<br>GCGCGGGGAACTCTCTAAAAGTATACATTTGTTCTTAAAGCATTTTTCCCATACAAACAACCCACCAACCTTAATGTAACA AAAGCCGGTTCAGG<br>GTCAGGTTCTGGTTCTGGAAGCGTT
**Yfp-CAR$^{\text{CR-II}}$ (MLS989)**	TTTACAGCTAGCTCAGTCCTAGGTATTATGCTAGCTACTAGAGAAAGAGGAGAAATACTAG**ATG**ACCATGATTACGCCAAGCTTGGTACCGAGCTCGGA<br>TCCACTAGTAACGGCCGCCAGTGTGCTGGAATTCGGCT CCGCAAACACAGGTTTATCCCCGCTGGCGCGGGGAACACATTACGCCTTTTTGCGATT<br>GCCCGGTTTTTGCCGGTTTATCCCCGCTGGCGCGGGGAACACTCTAAACATAACCTATTAT**TAA**TTAATGATTTTT**TAA**GCCAGTCACAATCTACCAAC<br>TTTATAGTATCA AAAGCCGGTTCAGGGTCAGGTTCTGGTTCTGGAAGCGTT
**Yfp-CAR$^{\text{CR-II}}$ Yfp positive control (1 bp inserted) (MLS990)**	TTTACAGCTAGCTCAGTCCTAGGTATTATGCTAGCTACTAGAGAAAGAGGAGAAATACTAG**ATG**ACCATGATTACGCCAAGCTTGGTACCGAGCTCGGA<br>TCCACTAGTAACGGCCGCCAGTGTGCTGGAATTCGGCT***C*** CCGCAAACACAGGTTTATCCCCGCTGGCGCGGGGAACACATTACGCCTTTTTGCGAT<br>TGCCCGGTTTTTGCCGGTTTATCCCCGCTGGCGCGGGGAACACTCTAAACATAACCTATTATTAATTAATGATTTTTTAAGCCAGTCACAATCTACCAA<br>CTTTATAGTATCA AAAGCCGGTTCAGGGTCAGGTTCTGGTTCTGGAAGCGTT

**Yfp-CAR$^{L-I, R-I}$ (MLS1000)**	TTTACAGCTAGCTCAGTCCTAGGTATTATGCTAGCTACTAGAGAAAGAGGAGAAATACTAG**ATG**ACCATGATTACGCCAAGCTTGGTACCGAGCTCGGA<br>TCCACTAGTAACGGCCGCCAGTGTGCTGGAATTCGGCTCA CGGTTTATCCCCGCTGGCGCGGGGAACTCTCTAAAAGTATACATTTGTTCT**TAA**AG<br>CATTTTTTCCCATACAAACAACCCACCAACCTTAATGTAACA AAAGCCGGTTCAGGGTCAGGTTCTGGTTCTGGAAGCGTT
**Yfp-CAR$^{L-II, R-I}$ (MLS1001)**	TTTACAGCTAGCTCAGTCCTAGGTATTATGCTAGCTACTAGAGAAAGAGGAGAAATACTAG**ATG**ACCATGATTACGCCAAGCTTGGTACCGAGCTCGGA<br>TCCACTAGTAACGGCCGCCAGTGTGCTGGAATTCGGCTCA CGGTTTATCCCCGCTGGCGCGGGGAACTCTCTAAACATAACCTATTAT**TAA**TTAAT<br>GATTTTT**TAA**GCCAGTCACAATCTACCAACTTTATAGTATCA AAAGCCGGTTCAGGGTCAGGTTCTGGTTCTGGAAGCGTT
**Yfp-CAR$^{L-I, R-II}$ (MLS1002)**	TTTACAGCTAGCTCAGTCCTAGGTATTATGCTAGCTACTAGAGAAAGAGGAGAAATACTAG**ATG**ACCATGATTACGCCAAGCTTGGTACCGAGCTCGGA<br>TCCACTAGTAACGGCCGCCAGTGTGCTGGAATTCGGCTCA CGGTTTATCCCCGCTGGCGCGGGGAACACTCTAAAAGTATACATTTGTTCT**TAA**AG<br>CATTTTTTCCCATACAAACAACCCACCAACCTTAATGTAACA AAAGCCGGTTCAGGGTCAGGTTCTGGTTCTGGAAGCGTT
**Yfp-CAR$^{L-II, R-II}$ (MLS1003)**	TTTACAGCTAGCTCAGTCCTAGGTATTATGCTAGCTACTAGAGAAAGAGGAGAAATACTAG**ATG**ACCATGATTACGCCAAGCTTGGTACCGAGCTCGGA<br>TCCACTAGTAACGGCCGCCAGTGTGCTGGAATTCGGCTCA CGGTTTATCCCCGCTGGCGCGGGGAACACTCTAAACATAACCTATTAT**TAA**TTAAT<br>GATTTTT**TAA**GCCAGTCACAATCTACCAACTTTATAGTATCA AAAGCCGGTTCAGGGTCAGGTTCTGGTTCTGGAAGCGTT

Supplementary table S2. Acquired spacers analyzed by SMRT sequencing.

See separate file.

Supplementary table S3. Oligonucleotides used in this study.

Name	Sequence (5' -> 3')	Description
LA007	TGTTACATTAAGGTTGGTGGGTTG	Anneals to CRISPR-I leader, used for spacer integration PCR.
LA112	AAACCAGTGAGTCATGAATGG	Anneals outside of CRISPR-array insertion after the ATG, used for spacer integration PCR.
LA120	ATTATTTGCACGGCGTCACACTTTG	Forward primer to amplify *araB::T7pol-tetA* from BL21AI for insertion in *araBAD* of BW25113.
LA121	**TGTGTTTTGGCAGCGCCAGGTTGGCTTCTAATACCCGGCG** TGGCGAATTGGGCCCTCTAG	Reverse primer to amplify *araB::T7pol-tetA* from BL21AI insertion in *araBAD* of BW25113. Homology in bold.
LA151	**GGTATTATGCTAGCTACTAGAGAAAGAGGAGAAATACTAG** ATGACCATGATTACGCCAAGC	Forward primer used for insert of Yfp-CAR in front of J23101 promoter in the *galK* locus. Homology in bold.
LA152	CTACTCAGGAGAGCGTTCACCG	Reverse primer used for insertion of Yfp-CAR in *galK* locus.
LA157	**GAGGTGTTACGTGGATATGTTGCTTATTACAAGTACTGCT** CCAAACTTTACCGCAATAATTTTCACTCCAGCGAAAAATT	Oligo used for scar-less deletion of CRISPR-II.
LA164	**GATCCACTAGTAACGGCCGCCAGTGTGCTGGAATTCGGCT** CCGCAAACACAGGTTTATC	Forward primer to amplify CRISPR-II from MG1655 to replace CRISPR-I in Yfp-CAR in MLS904. Homology in bold.
LA165	**GATCCACTAGTAACGGCCGCCAGTGTGCTGGAATTCGGCT** CCCGCAAACACAGGTTTATC	Forward primer to amplify CRISPR-II from MG1655 to replace CRISPR-I in Yfp-CAR in MLS904. Adds one extra base pair (underlined) to move the Yfp into frame.
LA166	**TAACGCTTCCAGAACCAGAACCTGACCCTGAACCGGCTTT** TGATACTATAAAGTTGGTAGATTGTGAC	Reverse primer to amplify CRISPR-II from MG1655 to replace CRISPR-I in Yfp-CAR in MLS904. Homology in bold.
LA168	AATTCAACTAAGATCGGTACTAC	Anneals in *yfp*, used for spacer integration PCR and for cloning of the minimal Yfp-CAR CRISPR arrays.
LA171	GGTTTATCCCCGCTGGCGCGGGGAACTCTCTAAA	Forward primer to amplify minimal CRISPR arrays.
LA172	GGTTTATCCCCGCTGGCGCGGGGAACACTCTAAA	Forward primer to amplify minimal CRISPR arrays.
LA173	G**ATCCACTAGTAACGGCCGCCAGTGTGCTGGAATTCGGCT** CACGGTTTATCCCCGCTG	Reverse primer to amplify minimal CRISPR arrays. Adds overhang for insertion by Lambda Red, homology in bold.
LML009	**TATTTATGAGCAGCATCGAAAAATAGCCCGCTGATATCAT**TGTA GGCTGGAGCTGCTTC	Forward Lambda Red primer to delete *cas3*-CRISPR-I. Homology in bold.
LML010	**TGATGGGTTTGAAAATGGGAGCTGGGAGTTCTACCGCAGA**CATA TGAATATCCTCCTTAG	Reverse Lambda Red primer to delete *cas3*-CRISPR-I. Homology in bold.

Supplementary table S4. Strains and plasmids used in this study.

Strain no.	Relevant genotype	Source / Description
MG1655	K-12 F⁻ λ⁻ *ilvG⁻ rfb-50 rph-1*	Wildtype strain
MLS640	BW25113 *araB::T7pol_tetA ΔaraA*	This study.
MLS902	MG1655 *araB::T7pol_tetA, ΔaraA, ΔCas3-CRISPR-I, ΔCRISPR-II, GalK::J23101_CRISPR-Iexp_link_SYFP2_opt*	This study. Wildtype strain with both CRISPR-Cas loci deleted. Also expresses the T7 polymerase. Yfp-CAR^{CR-I} expanded by 61 bp inserted in *galK* creating a positive control for Yfp fluorescence.
MLS904	MG1655 *araB::T7pol_tetA, ΔaraA, ΔCas3-CRISPR-I, ΔCRISPR-II, GalK::J23101_CRISPR-I_link_SYFP2_opt*	This study. As MLS902 but with unexpanded Yfp-CAR^{CR-I} inserted in *galK*.
MLS988	MG1655 *araB::T7pol_tetA, ΔaraA, ΔCas3-CRISPR-I, ΔCRISPR-II, GalK::J23101_kan-sacB_link_SYFP2_opt*	This study. As MLS904 but CRISPR-I array has been replaced by a *kan-sacB* cassette.
MLS989	MG1655 *araB::T7pol_tetA, ΔaraA, ΔCas3-CRISPR-I, ΔCRISPR-II, GalK::J23101_CRISPR-II_link_SYFP2_opt*	This study. As MLS904 but with Yfp-CAR^{CR-II}; an unexpanded CRISPR-II array instead of CRISPR-I.
MLS990	MG1655 *araB::T7pol_tetA, ΔaraA, ΔCas3-CRISPR-I, ΔCRISPR-II, GalK::J23101_CRISPR-II+1nt_link_SYFP2_opt*	This study. As MLS989 but with one base pair added to the CRISPR-II array moving *yfp* into frame, creating a positive control for Yfp fluorescence.
MLS1000	MG1655 *araB::T7pol_tetA, ΔaraA, ΔCas3-CRISPR-I, ΔCRISPR-II, GalK::J23101_L-I_R-I_link_SYFP2_opt*	This study. As MLS988 but *kan-sacB* cassette has been replaced by minimal CRISPR array, creating Yfp-CAR$^{L-I, R-I}$.
MLS1001	MG1655 *araB::T7pol_tetA, ΔaraA, ΔCas3-CRISPR-I, ΔCRISPR-II, GalK::J23101_L-II_R-I_link_SYFP2_opt*	This study. As MLS988 but *kan-sacB* cassette has been replaced by minimal CRISPR array, creating Yfp-CAR$^{L-II, R-I}$.
MLS1002	MG1655 *araB::T7pol_tetA, ΔaraA, ΔCas3-CRISPR-I, ΔCRISPR-II, GalK::J23101_L-I_R-II_link_SYFP2_opt*	This study. As MLS988 but *kan-sacB* cassette has been replaced by minimal CRISPR array, creating Yfp-CAR$^{L-I, R-II}$.
MLS1003	MG1655 *araB::T7pol_tetA, ΔaraA, ΔCas3-CRISPR-I, ΔCRISPR-II, GalK::J23101_L-II_R-II_link_SYFP2_opt*	This study. As MLS988 but *kan-sacB* cassette has been replaced by minimal CRISPR array, creating Yfp-CAR$^{L-II, R-II}$.
**Plasmids**	**Description**	**Source**
pCas1+2	Cas1 and Cas2 under the control of an IPTG-inducible T7 promoter. Streptomycin-resistant.	Yosef, I., Goren, M.G. & Qimron, U. Nucleic Acids Res 40, 5569-5576 (2012).
pCas1^{D221A}+2	As pCas1+2 but with a point mutation in Cas1 so integration is no longer observed.	Yosef, I., Goren, M.G. & Qimron, U. Nucleic Acids Res 40, 5569-5576 (2012).
pCSIR-T	Reporter plasmid where spacer integration leads to chloramphenicol resistance.	Díez-Villaseñor, C., Guzmán, N.M., Almendros, C., García-Martínez, J. & Mojica, F.J. RNA Biol 10, 792-802 (2013).
